# Divergent Effects of PERK and IRE1 Signaling on Cell Viability

**DOI:** 10.1371/journal.pone.0004170

**Published:** 2009-01-12

**Authors:** Jonathan H. Lin, Han Li, Yuhong Zhang, David Ron, Peter Walter

**Affiliations:** 1 Howard Hughes Medical Institute, University of California San Francisco, San Francisco, California, United States of America; 2 Department of Biochemistry and Biophysics, University of California San Francisco, San Francisco, California, United States of America; 3 Departments of Cell Biology and Medicine, Kimmel Center for Biology and Medicine, Skirball Institute, New York University School of Medicine, New York, New York, United States of America; UT MD Anderson Cancer Center, United States of America

## Abstract

Protein misfolding in the endoplasmic reticulum (ER) activates a set of intracellular signaling pathways, collectively termed the Unfolded Protein Response (UPR). UPR signaling promotes cell survival by reducing misfolded protein levels. If homeostasis cannot be restored, UPR signaling promotes cell death. The molecular basis for the switch between prosurvival and proapoptotic UPR function is poorly understood. The ER-resident proteins, PERK and IRE1, control two key UPR signaling pathways. Protein misfolding concomitantly activates PERK and IRE1 and has clouded insight into their contributions toward life or death cell fates. Here, we employed chemical-genetic strategies to activate individually PERK or IRE1 uncoupled from protein misfolding. We found that sustained PERK signaling impaired cell proliferation and promoted apoptosis. By contrast, equivalent durations of IRE1 signaling enhanced cell proliferation without promoting cell death. These results demonstrate that extended PERK and IRE1 signaling have opposite effects on cell viability. Differential activation of PERK and IRE1 may determine life or death decisions after ER protein misfolding.

## Introduction

Physiologic or pathologic processes that disturb protein folding in the endoplasmic reticulum (ER) activate a set of signaling pathways termed the Unfolded Protein Response (UPR). The molecular gatekeepers of the UPR are ER-resident transmembrane proteins that monitor the quality of protein folding in the ER and relay that information to the rest of the cell. In mammalian cells, PERK and IRE1 independently govern two key UPR signal transduction pathways [1]. PERK is a transmembrane kinase that phosphorylates translation initiation factor eIF2α, thereby reducing cellular protein synthesis and with it the load of proteins entering into the ER [2]. eIF2α phosphorylation also allows the translation of select mRNAs that contain small open reading frames in their 5′ untranslated regions, leading to the production of transcription activators, such as ATF4 and ATF5 [3], [4]. IRE1 is a bifunctional transmembrane kinase/endoribonuclease that induces the non-conventional splicing of *Xbp1* mRNA to produce another b-ZIP transcription activator, XBP1 [5]. In addition to splicing *Xbp1* mRNA, IRE1's kinase can also activate the c-Jun N-terminal kinase (JNK) signaling pathway through the MAP3K cascade [6], [7]. The transcription factors produced by PERK, IRE1, and other UPR signaling pathways collaborate to control behavior, metabolism, and ultimately cell fate in response to ER stress by inducing a wide array of targets that include protein folding chaperones such as *ERdj4* [8] and additional transcriptional activators such as *Chop* [3].

Genetic and pharmacological experiments have demonstrated that PERK signaling can confer both protective and proapoptotic effects in the face of ER stress. For instance, genetic deletion of *Perk* or impairment of *eIF2α* activity impaired cell survival [9], [10]. Conversely, transient artificial PERK activation or pharmacological eIF2α activation enhanced cell survival in response to ER protein misfolding [11], [12]. Deletion of downstream components of PERK signaling, *Atf4* and *Chop*, impaired or enhanced cell survival in response to protein misfolding depending on the cell type studied [3], [13], [14], [15], [16], [17].

Like PERK, IRE1 signaling has also been implicated in enhancing or impairing cell survival. Artificial extension of IRE1's RNAse function enhanced cell survival in the face of ER stress [18], [19]. RNAi knockdown of *Xbp1*, IRE1's RNAse target, impaired cell survival after protein misfolding in vitro and was required for the survival of multiple secretory cell types in vivo [20], [21]. Genetic deletion of *Ask1*, the MAP3K proposed to link IRE1 signaling to JNK, conferred resistance to ER stress-induced cell death [7], [22]. JNK can prevent or promote cell death depending on the specific stimuli, intensity, and/or duration of activation [23], [24], [25].

These findings demonstrate that PERK and IRE1 signaling can regulate cell survival after protein misfolding. How do cells modulate PERK and IRE1 activities to arrive at either cell fate? Previous studies demonstrated that the duration of PERK and IRE1 signaling varied markedly after the imposition of protein misfolding [18]. In particular, chronic ER stress led to IRE1 branch inactivation while PERK signaling was unaffected. These observations suggested that the progression toward cell death from unmitigated protein misfolding involved attenuation of IRE1 signaling coupled with persistent PERK activity. Previously, we employed chemical-genetic tools to artificially activate IRE1 and demonstrated a cytoprotective effect for its RNAse function in isogenic human cells [18]. Here, we employed a similar strategy to selectively activate PERK. We observed that sustained PERK signaling was detrimental to cell viability whereas the equivalent duration of IRE1 signaling was not, suggesting that extended PERK activity contributes to the cell death that occurs with chronic ER stress.

## Results

### Chemical-Genetic Control of PERK and IRE1 Signaling in Human Cells

**Figure 1:**
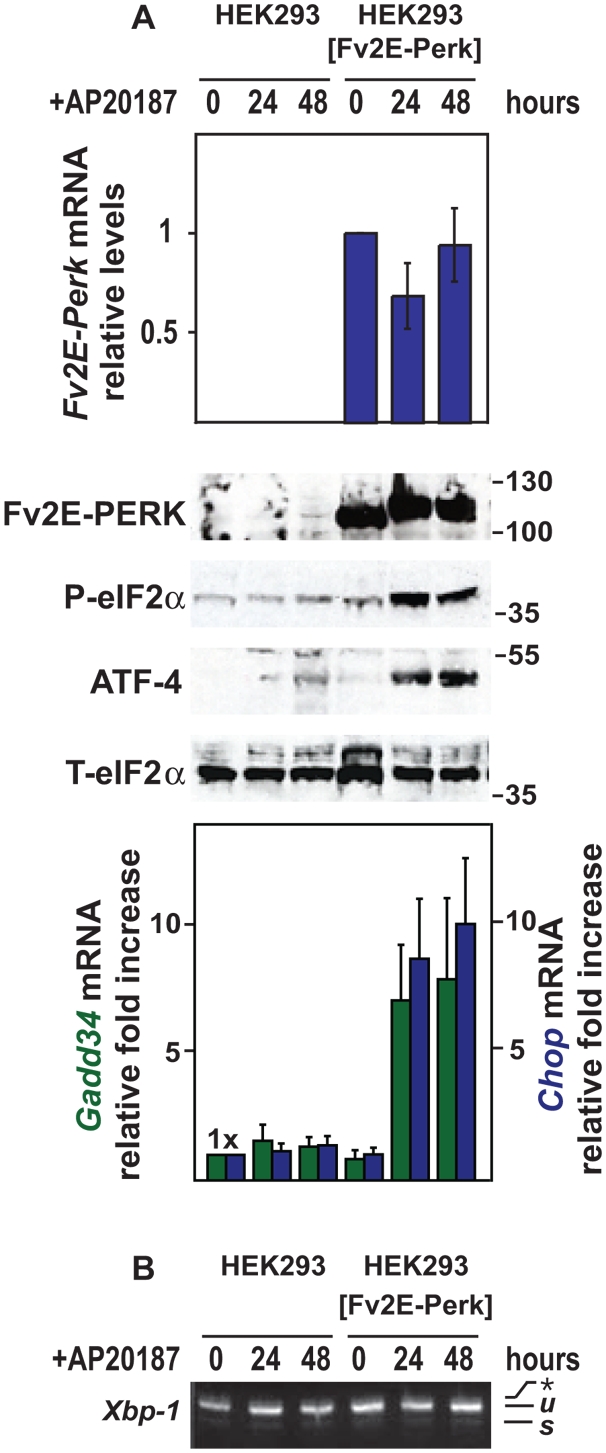
Selective and specific activation of PERK signaling. (A) Parental wild-type and transgenic HEK293 cells expressing the AP20187-sensitized *Fv2E-Perk* allele were treated for the indicated times with AP20187 (2 nM). *Fv2E-Perk*, *Gadd34*, and *Chop* mRNA levels were measured by quantitative PCR, normalized to levels of a housekeeping gene, *Rpl19*, and are shown relative to levels in untreated cells. Fv2E-PERK, phospho-eIF2α, and ATF4 proteins were detected by immunoblotting. Total eIF2α protein was measured as a loading control. (B) Parental wild-type and transgenic HEK293 cells expressing the AP20187-sensitized *Fv2E-Perk* allele were treated for the indicated times with AP20187 (2 nM). *Xbp1* mRNA splicing was assesed by RT-PCR. The unspliced (*u*) and spliced (*s*) *Xbp1* mRNA products are indicated as labeled. The asterisk indicates the position of a hybrid amplicon.

We previously used recombinase-directed site-specific DNA integration to introduce alleles into the genome of human embryonic kidney 293 (HEK293) cells [18]. This technique minimized perturbation of the native UPR as well as differences arising from position insertion variegation effects. We extended this strategy to create additional isogenic cell lines stably expressing an artificial PERK allele, *Fv2E-Perk*, which had previously been demonstrated to activate wild-type PERK signaling in hippocampal neurons and CHO cells upon addition of the dimerizing molecule, AP20187 [11], [26]. We observed stable *Fv2E-Perk* mRNA and protein expression at all times examined in our cells (Fig. 1A and Fig. S1). To determine how effectively we could recapitulate PERK branch signaling in HEK293 cells expressing *Fv2E-Perk*, we monitored multiple specific parameters of PERK activity after addition of the dimerizing agent, AP20187. After application of drug, we observed production of phosphorylated eIF2α and a downstream translational target ATF4 that approached levels seen with exposure to thapsigargin, an ER toxin that strongly induces all branches of the UPR (Fig. 1A and Fig. S1). Consistent with activation of these proximal parameters of PERK branch activity, we also observed increased mRNA levels of downstream PERK signaling transcriptional targets, *Chop* and *Gadd34*, after AP20187 application (Fig. 1A and Fig. S1). The GADD34 phosphatase has been demonstrated to target phosphorylated eIF2α and thereby deactivate PERK branch signaling [27]. Interestingly, we observed no diminution in phosphorylated eIF2α levels in the presence of AP20187, even though *Gadd34* was induced, suggesting that drug-activated Fv2E-PERK overcame the negative feedback effects of GADD34 on eIF2α (Fig. 1A). Lastly, to determine if AP20187's effects were confined to PERK or had non-specifically triggered ER stress, we examined a specific marker of IRE1 activation, splicing of *Xbp1* mRNA. Cells expressing Fv2E-Perk spliced *Xbp-1* mRNA in response thapsigargin, but no *Xbp1* mRNA splicing was observed at all concentrations and durations of AP20187 exposure that activated Fv2E-PERK (Fig. 1B and Fig. S1). Hence, these cells provide a system to examine the effects of selective PERK branch signaling.

**Figure 2:**
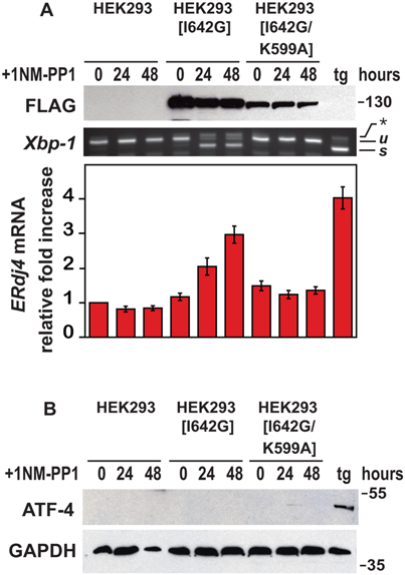
Selective and specific activation of IRE1 signaling. (A) Parental wild-type and transgenic HEK293 cells expressing the *Ire1[I642G]*, or *Ire1[I642G/K599A]* allele were treated for the indicated times with 1NM-PP1 (1 µM), and wild-type cells were treated for 4 hours with thapsigargin (tg) (300 nM). IRE1[I642G] and IRE1[I642G/K599A] protein was detected by immunoblotting for the FLAG epitope. GAPDH levels were assessed as a protein loading control. *Xbp1* mRNA splicing was determined by RT-PCR. The unspliced (*u*) and spliced (*s*) *Xbp1* mRNA products are indicated as labeled. *ERdj4* mRNA levels were measured by quantitative PCR, normalized to *Rpl19* mRNA levels, and are shown relative to levels in untreated cells. (B) Parental wild-type and transgenic HEK293 cells expressing the *Ire1[I642G]* or *Ire1[I642G/K599A]* alleles were treated for the indicated times with 1NM-PP1 (1 µM); wild-type cells were also treated for 4 hours with thapsigargin (tg) (300 nM). ATF4 protein was detected by immunoblotting. GAPDH levels were assessed as a protein loading control.

To study the effects of selective IRE1 branch activity on cell viability, we used transgenic HEK293 cells expressing an artificial *Ire1[I642G]* allele which we had previously shown could be regulated by addition of the ATP analogue, 1NM-PP1 [18], [19]. As a control for the specificity of IRE1[I642G]'s effects, we created additional cells expressing an allele of IRE1, Ir*e1[I642G/K599A]*, that bore a second missense mutation at amino acid position 599, which converted an essential lysine residue to alanine in the catalytic kinase domain of IRE1 [28]. We observed stable expression of IRE1[I642G] or IRE1[I642G/K599A] protein at all times examined in transgenic HEK293 cells (Fig. 2A). However, 1NM-PP1 application triggered *Xbp1* mRNA splicing and *ERdj4* mRNA induction, two parameters of IRE1 branch signaling, only in cells expressing IRE1[I642G], indicating that the additional K599A point mutation in IRE1[I642G/K599A] abolished its activity (Fig. 2A). To ascertain that 1NM-PP1's effects were confined to activation of IRE1[I642G], we examined a marker of PERK branch signaling, production of ATF4 protein. Parental and transgenic cells produced ATF4 in response to thapsigargin treatment, but no ATF4 protein was observed at all durations of 1NM-PP1 treatment that activated IRE1 signaling (Fig. 2B). Hence, these cells provided a system to examine the effects of selective IRE1 branch signaling on cell viability.

### Divergent Effects of Extended PERK and IRE1 Signaling on Cell Proliferation and Apoptosis

**Figure 3:**
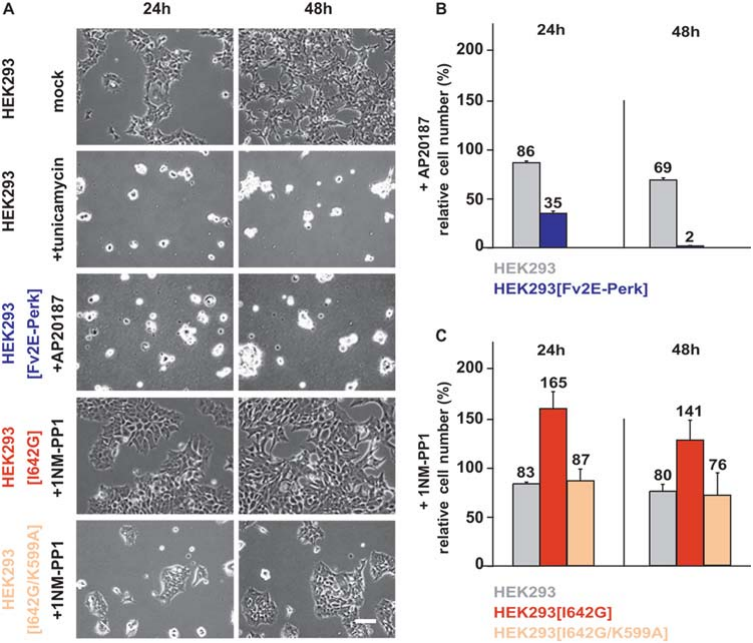
Sustained Perk signaling impairs cell proliferation. (A) Parental wild-type and isogenic HEK293 cells expressing *Fv2E-Perk*, *Ire1[I642G]*, or *Ire1[I642G/K599A]* alleles were treated with tunicamycin (5 µg/ml), 1NM-PP1 (1 µM), or AP20187 (2 nM), videographed for 48 hours, and frames from indicated time points are shown. Magnification bar, 125 µm. (B) Parental wild-type and transgenic HEK293 cells expressing the *Fv2E-Perk* allele were treated with AP20187 (2 nM), counted, and are shown relative to numbers of mock-treated cells at the indicated times. (C) Parental wild-type and transgenic HEK293 cells expressing *Ire1[I642G]* or *Ire1[I642G/K599A]* alleles were treated with 1NM-PP1 (1 µM), counted, and are shown relative to numbers of mock-treated cells at the indicated times.

**Figure 4:**
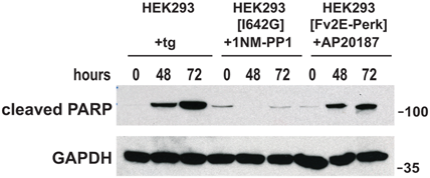
Sustained Perk signaling promotes apoptosis. Parental wild-type and isogenic HEK293 cells expressing *Ire1[I642G]* or *Fv2E-Perk* were treated with thapsigargin (300 nM); 1NM-PP1 (1 µM); or AP20187 (2 nM) for the indicated times. Cleaved PARP protein was assessed by immunoblot. GAPDH protein levels served as a loading control.

We used these isogenic cell lines bearing *Fv2E-Perk*, *Ire1[I642G]*, or *Ire1[I642G/K599A]* to address how selective IRE1 or PERK signaling affected cell viability with respect to proliferation and apoptosis. Chronic protein misfolding induced by multi-day exposure to tunicamycin or thapsigargin severely impaired cell proliferation and triggered apoptosis in wild-type cells (Video S1, Fig. 3A, Fig. 4). When we selectively activated PERK signaling in cells bearing *Fv2E-Perk* by application of AP20187 for up to 48 hours, we observed a pronounced reduction in cell numbers compared to mock treated or parental cells exposed to AP20187 (Video S2, Fig. 3A, Fig. 3B). By contrast, when we selectively activated IRE1 signaling in transgenic cells bearing *Ire1[I642G]* by application of 1NM-PP1, we observed increased cell numbers compared to mock treated or parental cells exposed to the drug (Video S3, Fig. 3A, Fig. 3C). The advantage in proliferation specifically required functional IRE1 branch activity, since 1NM-PP1 exposure did not enhance survival in cells bearing the doubly-mutated *Ire1[I642G/K599A]* allele (Fig. 3A, Fig. 3B). In sum, these studies clearly demonstrated that sustained PERK signaling impairs cell proliferation while IRE1 signaling promotes cell growth.

We also observed striking morphologic changes in cells, in which PERK signaling was selectively activated, including retraction of cellular extensions, loss of refractiveness under phase-contrast microscopy, and detachment from the underlying matrix (Video S2, Fig. 3A). These physical changes resembled those seen in cells undergoing cell death after exposure to lethal concentrations of ER stress-inducing agents, such as tunicamycin or thapsigargin (Video S1, Fig. 3A). By contrast, none of these morphologic changes were seen in cells in which IRE1 branch signaling had been selectively activated (Video S3, Fig. 3A). These morphologic changes suggested that sustained PERK activity triggered apoptosis in addition to impairing cell proliferation.

To determine if molecular markers of apoptosis occurred in these cells, we next examined cleavage of poly(ADP-ribose) polymerase (PARP), a nuclear DNA repair enzyme that undergoes proteolysis in response to many apoptotic stimuli [29]. Robust production of cleaved PARP was observed after 48 to 72 hours of exposure to tunicamycin or thapsigargin (Fig. 4 and Fig. S2). Minimal PARP cleavage was seen in wild-type cells exposed to 1NM-PP1 or AP20187, indicating that these small molecules did not trigger cell death at bio-efficacious concentrations (Fig. S2). When AP20187 was applied to cells expressing Fv2E-PERK, we saw strong production of cleaved PARP (Fig. 4). By contrast, when 1NM-PP1 was added to cells expressing IRE1[I642G], PARP was not cleaved (Fig. 4). Taken together with the cytomorphologic changes, these findings indicate that sustained PERK signaling triggers apoptosis, whereas IRE1 signaling does not when activated for equivalent duration. Consistent with the incompatibility of extended PERK signaling with viability, loss of the Fv2E-PERK transgene was observed in all cells that were able to proliferate in the presence of AP20187 (Fig. S3).

## Discussion

The UPR detects and responds to ER protein misfolding acutely by enhancing the protein folding capacity of the ER, but, if protein misfolding persists, the UPR promotes cell death. The molecular basis for this switch between protective and proapoptotic UPR function is poorly understood. Prior studies from our group had delineated distinct molecular phases of UPR signaling in which acute ER stress activated both PERK and IRE1, but persistent chronic ER stress activated only the PERK pathway and attenuated IRE1 signaling [18]. These observations led to the hypothesis that the switch in IRE1 signaling coupled with unabated PERK activity contributed to the transition from protective to proapoptotic UPR function. To examine this model, we used chemical-genetic approaches to activate PERK or IRE1 in isolation in isogenic “sister” human cell lines and observed that chronic PERK signaling promotes cell death. By contrast, IRE1 activity enhances cell survival. Coupled with our previous studies, these findings provide compelling evidence that the time course of PERK and IRE1 signaling plays a critical role in determining how the UPR selects between life and death cell fates.

Our current finding that chronic PERK activity impairs cell viability is consistent with our prior study showing that selective activation of PERK triggered cell death in other cell types [11] as well numerous reports demonstrating that the CHOP transcription factor, produced by PERK signaling, actively promotes apoptosis in vitro and in vivo [3], [13], [15], [16]. How can this proapoptotic capacity of PERK signaling be reconciled with its ability to enhance cell survival in the face of protein misfolding [9], [10], [11], [12]? Our findings suggest that the duration and/or strength of PERK signaling may determine whether cytoprotective or proapoptotic outcomes predominate. In our model, transient PERK signaling protects cells by temporarily dampening cellular protein synthesis and thus reducing misfolded protein levels in the ER. Transient PERK signaling may also be insufficient to induce CHOP levels to proapoptotic threshholds, given *Chop's* inherent mRNA and protein instability [30]. However, persistent PERK signaling could ultimately impair cell viability if extended translational inhibition interrupted the generation of proteins vital for cellular homeostasis. Persistent PERK signaling could also lead to the accumulation of sufficient CHOP to drive cell death. Intriguingly, in some cell types, CHOP directly induces the transcription of *Bim*, a proapoptotic member of the BCL2 protein family that directly elicits cell death by permeabilizing the mitochondrial outer membrane [31]. A PERK-CHOP-BIM signaling axis could link chronic protein misfolding in the ER to activation of the intrinsic apoptosis machinery in the mitochondria. Additional parallel proapoptotic signaling pathways must also exist given the continued sensitivity of *Perk* and *Chop* null cells to ER protein misfolding [9], [13].

Can IRE1 also transmit apoptotic signals from the ER? While we demonstrate a cytoprotective function for IRE1 signaling through its RNAse activity, in mammalian cells, IRE1 has acquired additional properties independent of splicing that include activation of the JNK signaling pathway and selective biochemical interactions with the BAK and BAX proteins of the BCL2 family of apoptotic regulators [6], [32]. The JNK signaling pathway and BCL2 proteins are key regulators of cell survival and apoptosis in response to numerous stimuli [23], [33]. Although the consequences of their interactions with the IRE1 signaling pathway on cell survival are unknown, they raise the possibility that IRE1 employs multiple downstream modules besides XBP1 generation to regulate cell fate after activation by protein misfolding. IRE1's oligomerization status has recently been shown to regulate its RNAse activity [34]. Investigating the effect of IRE1 polymerization status on JNK and BAX/BAK activity may shed additional insight into IRE1's effects on cell survival.

Divergent effects of persistent PERK and IRE1 signaling on cell proliferation and survival may also underlie the phenotypes observed in several pathologic and physiological situations in vivo. Mice on high-fat diets developed hepatocyte steatosis, accompanied by inflammation and PERK activation, suggesting a link between PERK signaling and cellular dysfunction [35], [36]. By contrast, selective expression of spliced XBP1 protein in B-cells dramatically enhanced cell numbers, leading to a multiple myeloma-like phenotype [37], consistent with the ability of IRE1's RNAse function to promote cell proliferation and survival. Pharmacological modulation of PERK or IRE1 signaling could provide new approaches to treat diseases associated with ER stress.

## Materials and Methods

### Molecular Biology

Generation of the AP20187 dimerizable *Fv2E-Perk* allele and 1NM-PP1 sensitized *Ire1[I642G]* allele has been previously described (Lu et al., 2000; Lin et al., 2007). To construct the *Ire1[I642G/K599A]* allele, QuikChange site-directed mutagenesis (Stratagene, San Diego, CA) was used to insert a lysine to alanine missense mutation in the *Ire1[I642G]* allele at amino acid position 599.

RT-PCR analysis of *Xbp1* mRNA splicing was performed as previously described (Lin et al., 2007). Primers used for quantitative PCR analysis included: *Fv2E-Perk* mRNA, 5′- TGAGTGTGGGTCAGAGAGCCAAAC-3′ and 5′- ACGGAGTCGTATTTACTTTCAGTC-3′; human *Rpl19* mRNA, 5′-ATGTATCACAGCCTGTACCTG–3′ and 5′-TTCTTGGTCTCTTCCTCCTTG-3′; human *Chop* mRNA, 5′-ACCAAGGGAGAACCAGGAAACG-3′ and 5′-TCACCATTCGGTCAATCAGAGC-3′; human *Gadd34* mRNA, 5′- CCTCTACTTCTGCCTTGTCTCCAG -3′ and 5′- TTTTCCTCCTTCTCCTCGGACG -3′; and human *ERdj4* mRNA, 5′- TGGTGGTTCCAGTAGACAAAGG-3′ and 5′- CTTCGTTGAGTGACAGTCCTGC-3′. Quantitative PCR was performed using a MJ Opticon 2 DNA Engine (Bio-Rad, Hercules, CA) as previously described (Lin et al., 2007).

### Protein Analysis

The following antibodies and dilutions were used for Western analyses: anti-FKBP at 1∶1000 (Affinity BioReagents, Golden, CO); anti-eIF2α at 1∶2000 (Cell Signaling, Natick MA); anti-phospho-eIF2α at 1∶500 (Cell Signaling, Natick, MA); anti-ATF4 at 1∶2000 (Santa Cruz Biotechnologies, Santa Cruz, CA); anti-GAPDH at 1∶10000000 (AbCAM, Cambridge, MA); anti-FLAG at 1∶5000 (Sigma, St. Louis, MO); and anti-PARP at 1∶2000 (Cell Signaling, Natick, MA).

### Cell Culture

HEK293 cell lines were maintained at 37°C, 5% CO2 in DMEM media supplemented with fetal calf serum, glutamine, and antibiotics (Invitrogen, San Diego, CA). Tunicamycin and thapsigargin were obtained from Calbiochem EMD Bioscience Inc. (Darmstadt, Germany). AP20187 was provided by Ariad Pharmaceuticals (Cambridge, MA) and used as directed. 1NM-PP1 was used as previously described [18]. The *Fv2E-Perk*, *Ire1[I642G]*, and *Ire1[I642G/K599A]* alleles were integrated into HEK293 cells bearing *frt* sites as previously described (Lin et al., 2007). Multiple independent isogenic clones were analyzed with identical findings.

The CHO cell line bearing *Fv2E-Perk* has been previously described [26]. To obtain resistant cells, CHO cells bearing *Fv2E-Perk* were plated at clonal density and grown for 10 days in 100 nM AP20187 (and 3 µg/ml puromycin to enforce expression of the *Fv2E-Perk* retroviral transgene). Multiple resistant clones were identified under such conditions and individually expanded for Fv2E-PERK protein expression analysis.

### Cell Microscopy, Image Acquisition, and Cell Counts

Wild-type or isogenic HEK293 cells bearing *Fv2E-Perk*, *Ire1[I642G]*, or *Ire1[I642G/K599A]* alleles were plated at densities of 75000 cells/ml and live-cell imaging was performed using an inverted microscope (Nikon TE2002E2) with a 10× 0.3NA objective and a cooled charge-coupled device camera (Coolsnap HQ2, Photometrics) in a sealed humidified 5% CO2, 37 C chamber. Images were acquired at 5-minute intervals for 48 hours after application of tunicamycin (5 µg/ml), 1NM-PP1 (1 µM), AP20187 (2 nM), or dimethylformamide solvent using Nikon Imaging Systems Elements 2.3 software. Images were exported as TIFF files into ImageJ software to compile into video files and to capture frames for cell counts. Three to six independent imaging experiments were conducted for each condition, and representative videos are shown.

## Supporting Information

Figure S1 Titration of AP20187 on HEK293 cells expressing Fv2E-Perk. (A) Transgenic HEK293 cells were treated for 4 hours with thapsigargin (300 nM) or the indicated concentrations of AP20187. Fv2E-PERK and ATF4 proteins were detected by immunoblotting. Chop mRNA levels were measured by quantitative PCR, normalized to Rpl19 mRNA levels, and shown relative to levels in mock-treated cells. (B) Transgenic HEK293 cells were treated for 4 hours with thapsigargin (300 nM) or the indicated concentrations of AP20187. Xbp1 mRNA splicing was assesed by RT-PCR. The unspliced (u) and spliced (s) Xbp1 mRNA products are indicated as labeled.
(1.67 MB EPS)

Figure S2 Effect of tunicamycin, 1NM-PP1, or AP20187 on PARP processing in HEK293 cells. Parental wild-type HEK293 cells were treated for the indicated times with tunicamycin (tu) (5 Î¼g/ml), 1NM-PP1 (1 Î¼M), or AP20187 (2 nM). Cleaved PARP protein was assessed by immunoblot. GAPDH protein levels served as a loading control.
(1.94 MB EPS)

Figure S3 Loss of Fv2E-PERK restores cell viability in CHO cells. Fv2E-PERK protein (+/− phosphorylation) was examined by immunoblotting in parental CHO cells expressing stably-integrated Fv2E-Perk and 6 clonal derivatives that grew in the presence of AP20187 (100 nM). Ponceau S staining of the immunoblot revealed equivalent protein levels and served as a loading control (data not shown). Where indicated, cells were exposed to AP20187 (100 nM) for 30 minutes.
(0.63 MB EPS)

Video S1 HEK293 cells treated with mock solvent (left frame) or tunicamycin (right frame) for 48 hours.
(7.81 MB MOV)

Video S2 HEK293 cells expressing Fv2E-PERK treated with mock solvent (left frame) or AP20187 (right frame) for 48 hours.
(10.37 MB MOV)

Video S3 HEK293 cells expressing IRE1[I642G] treated with mock solvent (left frame) or 1NM-PP1 (right frame) for 48 hours.
(10.15 MB MOV)
